# Melanonychia

**DOI:** 10.1155/2012/952186

**Published:** 2012-06-27

**Authors:** Julie Jefferson, Phoebe Rich

**Affiliations:** Oregon Health and Science University, Portland, OR, USA

## Abstract

Melanonychia, or melanin-derived brown-to-black nail pigmentation, is a diagnostic challenge for clinicians. The most serious disease of the nail unit, melanoma, primarily presents with melanonychia. However, melanonychia most often occurs as a result of benign etiologies such as nail matrix melanocytic activation, nail matrix melanocytic hyperplasia, and nail invasion by melanin-producing pathogens. Regrettably, patients with nail apparatus melanoma are often initially misdiagnosed, and due to diagnostic delays of an average of 2 years, melanoma of the nail unit carries a poor prognosis. Having a thorough knowledge of the various causes of melanonychia and using a systematic approach when evaluating brown-to-black nail pigmentation may help prevent misdiagnosis and thereby improve prognosis.

## 1. Introduction

Roughly two-thirds of cases of nail apparatus melanoma (NAM) are characterized by melanin-derived brown-to-black nail pigmentation, or melanonychia [1]. However, melanonychia is an ambiguous clinical finding that most commonly occurs as a result of benign etiologies such as nail matrix melanocytic activation, nail matrix melanocytic hyperplasia, and nail invasion by melanin-producing pathogens. Additionally, other nail pathogens, exogenous substances, and subungual hemorrhage can cause non-melanic brown-to-black nail pigmentation [1–3]. Unfortunately, patients with NAM are often initially misdiagnosed [2]. Due to diagnostic delays of an average of 2 years, NAM carries a poor prognosis with reported 5-year and 10-year survival rates of 30% and 13%, respectively [4, 5]. Having a thorough knowledge of the various causes of brown-to-black nail pigmentation and using a systematic approach when evaluating clinical cases may help prevent misdiagnosis and ultimately save a life. 

## 2. Melanonychia 

Melanin within the nail plate is usually produced by nail matrix melanocytes [2, 6]. Active melanocytes transfer melanin-rich melanosomes by way of dendrites to differentiating nail matrix-derived onychocytes [2, 6]. This process most commonly results in a longitudinal band of melanonychia, but total or transverse melanonychia may also occur, although rarely [2, 6, 7]. Melanonychia usually originates in the distal nail matrix [2, 6]. Most melanocytes in the proximal nail matrix lie dormant in the lower 2–4 germinative cell layers while active (as well as dormant) melanocytes exist in the 1st and 2nd germinative layers of the distal nail matrix [2, 6]. The etiologies of melanonychia may be divided into 2 broad categories: melanocytic activation and melanocytic hyperplasia (Table 1) [1–3]. 

### 2.1. Melanocytic Activation

Melanocytic activation (also termed melanocytic stimulation or functional melanonychia) describes the process by which melanonychia results from increased melanic pigmentation of the nail matrix epithelium and nail plate without a concurrent increase in the number of melanocytes [2]. Seventy-three percent of adult cases of single digit longitudinal melanonychia (LM) occur as a result of melanocytic activation [2, 8]. Several physiologic, local and regional, dermatologic, systemic, iatrogenic, and syndromic factors may lead to melanocytic activation [2, 9].

**Physiologic Causes**

**Figure 1:**
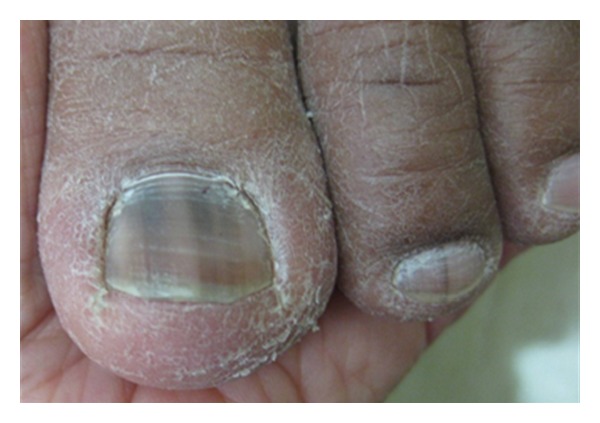
Racial melanonychia.

Physiologic causes of LM include racial melanonychia and pregnancy (Figure 1) [2]. Darkly pigmented individuals such as blacks, Asians, Hispanics, and Middle Easterners frequently have benign longitudinal pigmented bands [2, 4]. The number and width of the bands increases with age [2, 10]. In fact, nearly 100% of blacks develop 1 or more pigmented bands by the age of 50 years [2]. Pigmented bands are most often located in the digits used for grasping such as the thumb, index finger, and middle finger, or in those digits prone to trauma such as the great toe [2]. Additionally, the nail plates of patients with darker phototypes may exhibit LM more easily with other causes of melanocytic activation [2].

**Local and Regional Causes**

**Figure 2:**
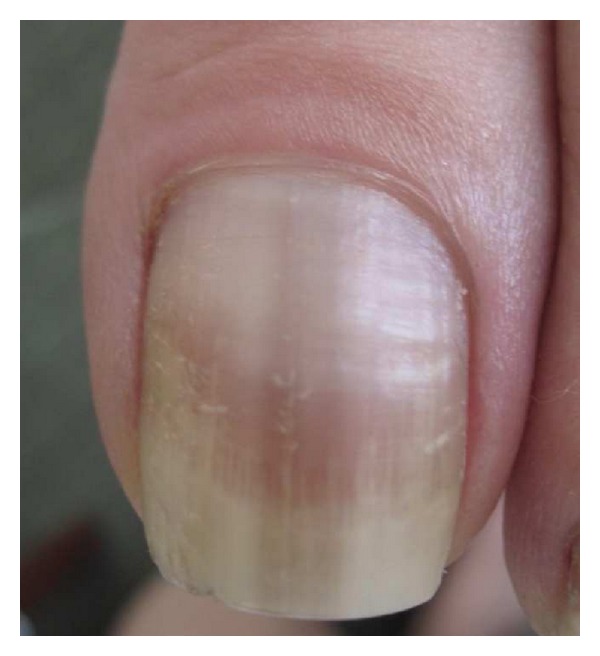
Traumatic melanonychia following a fracture of the great toe.

If melanonychia is associated with abnormalities of the nail plate or the periungual tissues, the possibilities of onychotillomania, nail biting, frictional trauma, and even carpal tunnel syndrome should be explored (Figure 2) [2, 11]. If melanonychia is symmetric and affects the lateral and external part of the 4th or 5th toenail and great toe, repeated trauma from ill-fitting shoes or overriding toes is a likely cause [2, 12].

**Dermatologic Causes**

Inflammation due to dermatologicconditions such as onychomycosis, paronychia, psoriasis, lichen planus, amyloidosis, and chronic radiodermatitis may lead to the activation of melanocytes and subsequently the appearance of a light-brown band [2]. Oftentimes melanonychia occurs following the resolution of the inflammatory process [2]. Nonmelanocytic tumors including onychomatricoma [13], Bowen's disease [14], myxoid pseudocyst [10], basal cell carcinoma [10], subungual fibrous histiocytoma [10], verruca vulgaris [10], and very rarely subungual linear keratosis [7, 15] have also been documented to cause melanocytic activation resulting in LM [2].

**Systemic Causes**

Melanonychia due to systemic causes often manifests as multiple bands involving both fingernails and toenails. Interestingly, melanonychia associated with Addison's disease, nutritional disorders, and AIDS is commonly accompanied by cutaneous and mucosal pigmentation [2, 16]. Melanonychia has been reported to be due to alcaptonuria, hemosiderosis, hyperbilirubinemia, and porphyria [2, 10].

**Iatrogenic Causes**

**Figure 3:**
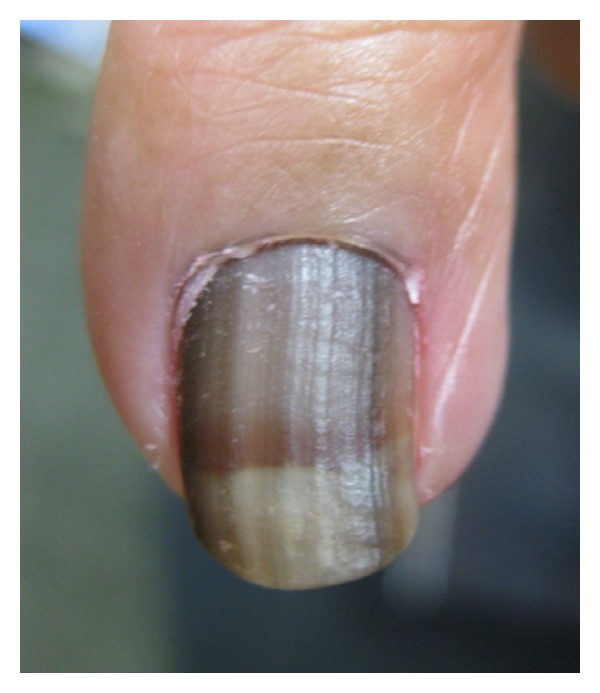
Drug-related melanonychia secondary to hydroxyurea-induced melanocytic activation.

Iatrogenic causes of melanocytic activation include medications (especially chemotherapeutic agents) [17–19], phototherapy, X-ray exposure, and electron beam therapy [20] (Figure 3, Table 2) [2]. Presentations of iatrogenically-induced melanonychia may vary significantly depending on the exposure, but are usually associated with melanonychia of several fingernails and toenails [2]. Fortunately when melanonychia is drug-related, it typically fades slowly following drug withdrawal [2]. Transverse melanonychia, while uncommon, is most commonly reported as a result of iatrogenic causes. Transverse melanonychia has occurred in conjunction with use of the following: electron beam therapy [20], conventional radiographic therapy to treat hand dermatitis (used in the 1950s and 1960s) [21, 22], psoralen with ultraviolet A (PUVA) [23–26], infliximab [27], zidovudine [28], prolonged antimalarial therapy with amodiaquine, chloroquine, mepacrine, or quinacrine [2, 26, 28], and chemotherapy with agents such as doxorubicin, bleomycin, cyclophosphamide, daunorubicin, dacarbazine, 5-fluorouracil, methotrexate [20], and hydroxyurea [26, 29, 30]. Transverse melanonychia associated with electron beam therapy and PUVA is benign and typically resolves with the cessation of treatment [20, 23, 26]. Interestingly, for antimalarials amodiaquine, chloroquine, and mepacrine, transverse melanonychia may be attributable to either melanin production or more commonly ferric dyschromia [2].

**Syndrome-Associated Melanonychia**

Syndrome-associated melanonychia, which occurs in conjunction with Laugier-Hunziker, Peutz-Jeghers, and Touraine syndromes, typically involves multiple digits, and all of these syndromes are also characterized by mucosal pigmented macules involving the lips and oral cavity [2]. Laugier-Hunziker syndrome is a chronic benign mucocutaneous syndrome that generally arises spontaneously in 20–40-year-old Caucasian adults, while Peutz-Jeghers and Touraine syndromes are autosomal dominantly inherited disorders that typically manifest during childhood and are associated with intestinal polyposis and an increased risk for gastrointestinal and pancreatic malignancies [2].

### 2.2. Melanocytic Hyperplasia

**Figure 4:**
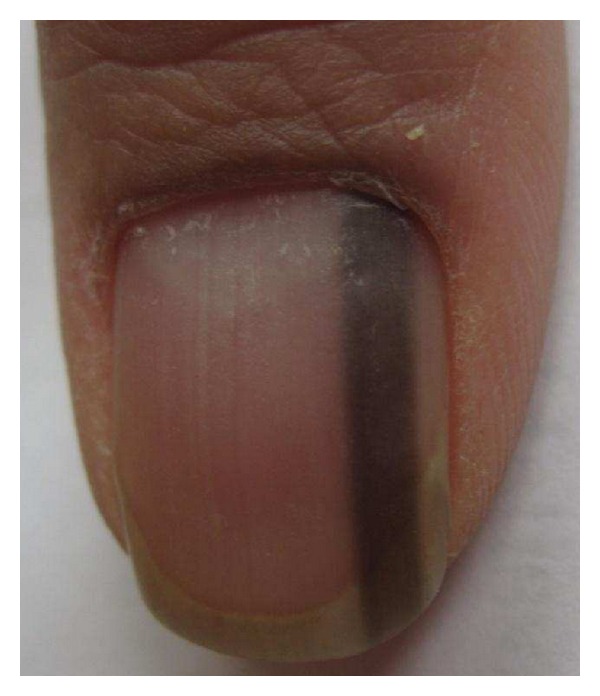
Nail apparatus lentigo in an adult.

**Figure 5:**
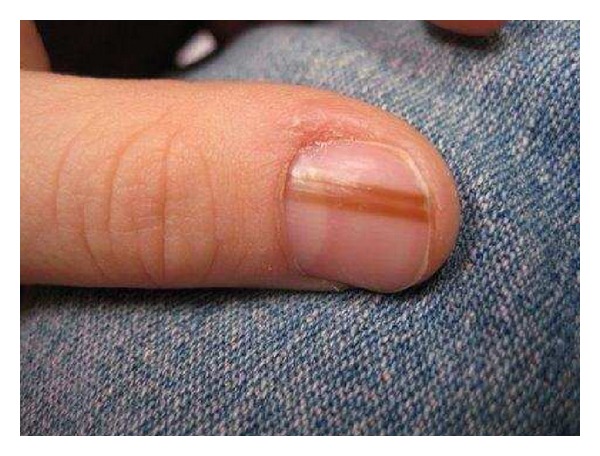
Nail apparatus nevus in a child.

The 2nd broad category of melanonychia, melanocytic hyperplasia, is characterized by an increase in the number of matrix melanocytes [2]. Both benign and malignant forms exist [2]. Benign melanocytic hyperplasia is subdivided into 2 categories, lentigines, when nests of melanocytes are absent, and nevi, when at least 1 melanocytic nest is present [2]. While lentigines are observed more often than nevi in adults, nevi are found far more often than lentigines in children (Figures 4 and 5). NAM occurs rarely in children, and benign melanocytic hyperplasia constitutes 77.5% of cases of childhood melanonychia [2, 31]. 

Nail apparatus nevi primarily involve the fingernails, with the thumbnail being most commonly affected. Nevi can be congenital or acquired, and the majority are junctional [1, 2, 8, 31, 32]. One half of cases of nevi are characterized by a bandwidth of over 3 mm, two-thirds by melanic brown-black pigmentation, and one-third by melanic periungual pigmentation [2]. Nevertheless, nail matrix nevi may also present as scarcely pigmented bands [2, 8, 9, 31, 32]. 

**Figure 6:**
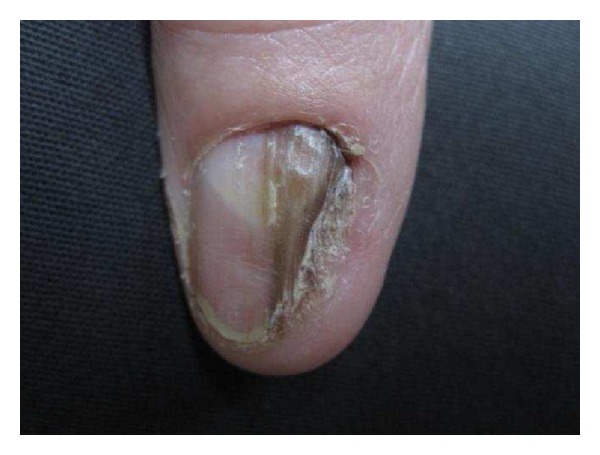
Nail apparatus melanoma *in situ* in a middle-aged Caucasian adult.

Malignant melanocytic hyperplasia includes both *in situ* and invasive melanoma of the nail apparatus (Figure 6) [2]. Melanoma is most commonly observed in the thumbs, index fingers, and great toes in patients mean age 60–70 years [2]. Roughly, 1–3% of melanomas in Caucasians [1, 33–35], 15–20% in blacks [1], 16% in Mexicans [1, 36], 10–30% in Japanese [1, 37, 38], 17% in Chinese [1, 39], and 33% in Native Americans [1, 40] occur within the nail unit [4, 10]. Because other forms of melanoma occur less often in people with darker phototypes compared to Caucasians, the absolute incidence of nail-associated melanoma is similar among the various racial groups [1, 2, 41]. 

### 2.3. Pathogen-Induced Melanonychia

As previously highlighted, certain pathogens involved in onychomycosis or paronychia can trigger an inflammatory response that induces melanocytic activation resulting in melanonychia. In addition, some Gram-negative bacterial pathogens such as *Proteus mirabilis* [42], and some dermatophyte strains, such *as trichophyton rubrum.* *Var nigricans,* can produce melanin and infrequently present as a linear streak [1]. Several other organisms can present with linear brown-to-black nail dyschromia by producing a pigment other than melanin. 

## 3. Clinical Evaluation of Melanonychia

### 3.1. History

A thorough history with particular attention to the onset, progression, and possible triggers of melanonychia should be obtained. Providing patients with a nail questionnaire with inquiries concerning their occupation, hobbies, exposure to topical substances, history of digital trauma, drug history, medical history, and family history prior to the visit allows them ample time to prepare thoughtful answers, and also allows the physician more time during the appointment to focus on key portions of the history. Nail apparatus melanoma should be suspected in any patient with unexplained melanonychia who provides a history containing any of the following features:

- involvement of a single digit (especially the thumb, index finger, or great toe);
- development during the fourth decade of life or later;
- development in the setting of a history of digital trauma;
- development in the setting of a personal/family history of melanoma or dysplastic nevus syndrome;
- development in the setting of nail dystrophy;
- abrupt development or change (darkening or widening proximally) [1, 4].

### 3.2. Physical Examination

Initial physical examination should include evaluation of all twenty nails, skin, and mucous membranes while keeping in mind all potential causes of brown-to-black nail pigmentation. Questions to help guide the initial examination of the nails include the following:

- Are one or more nails involved?
- If multiple nails are involved, is one particular nail changing or different from the rest?
- Is the discoloration located on top of, within, or beneath the nail plate?
- Is the discoloration linear in orientation?
- Is the band wider or darker proximally?
- Is the discoloration associated with nail plate dystrophy?

**Exogenous Substances**

The possibility of linear nail pigmentation due to an exogenous substance on top of or beneath the nail plate should be ruled out. Notably, a linear band secondary to pigment production by a nail pathogen is typically wider distally than proximally indicating a distal rather than proximal (or matrix) origin [1]. Infection can be confirmed by histopathologic examination, and/or culture [1].

Exogenous substances such as dirt, tar, tobacco, and potassium permanganate are usually located on top of the nail plate and follow the shape of the proximal nail fold rather than the lunula [1, 2]. Exogenous substances grow-out with the nail plate, and can sometimes be simply scraped off [1, 2]. In cases of suspected potassium permanganate staining, one component, manganese dioxide, can be reduced to a colorless compound with the application of 5–10% ascorbic acid [1].

Occasionally, subungual hematomas can present in linear fashion [1]. In such instances, dermoscopy may be used to help distinguish blood from melanin and is discussed later. Direct visualization of the underlying nail bed by punching a hole in the nail plate in the area of dyschromia can be performed to confirm its presence [2, 43]. Additionally, blood is also characterized by a positive pseudoperoxidase reaction (Hemostix test) [1].

**Hutchinson's Sign**

**Figure 7:**
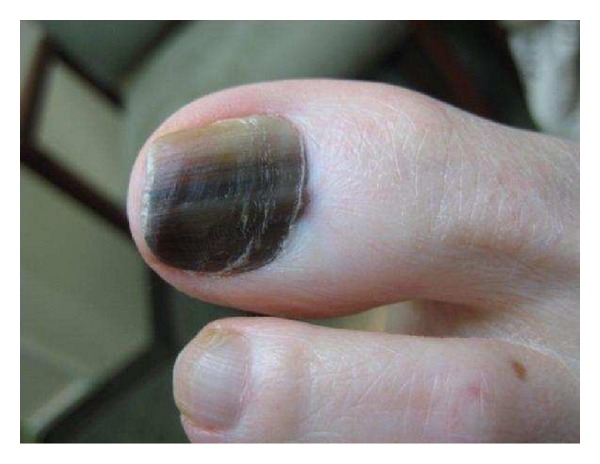
Hutchinson's sign in a nail apparatus melanoma *in situ*.

**Figure 8:**
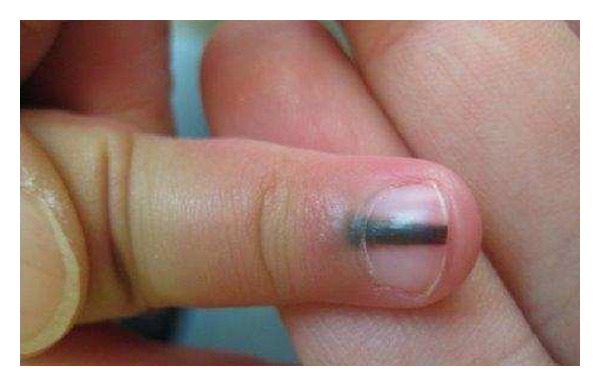
Pseudo-Hutchinson's sign in a nail apparatus nevus.

The surrounding skin should be carefully examined for discoloration similar to that seen in the nail plate. Hutchinson's sign, or the extension of pigment from the matrix to the perionychium in association with NAM is sometimes helpful in confirming the clinical diagnosis but is an inconsistent feature (Figure 7) [4]. Melanoma can occur without Hutchinson's sign. Moreover, pseudo-Hutchinson's sign, or the presence or illusion of pigment in the perionychium, is associated with both benign and malignant conditions in the absence of melanoma (Figure 8) [2, 4, 44].

**Dermoscopy**

**Figure 9:**
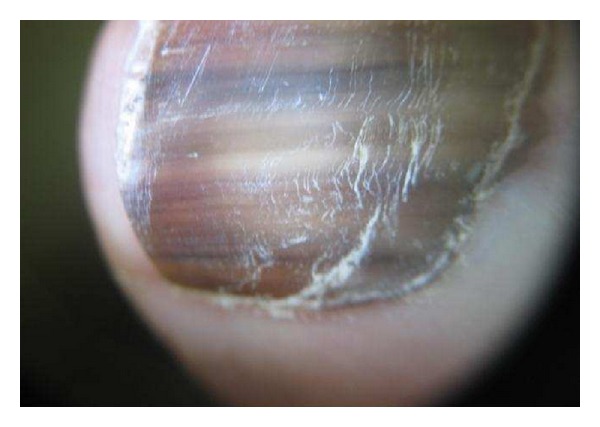
Dermoscopy of the nail apparatus melanoma *in situ* seen in Figure 7. Notice the brown coloration of the background and the presence of irregular longitudinal lines per color, spacing, thickness, and parallelism.

Dermoscopy may provide clues when deciding whether biopsy is necessary [3, 43]. However, the topic of dermoscopic findings associated with NAM versus benign etiologies is currently controversial among experts and further studies are needed. Blood spots are usually purple to brown in color, and are characterized by well-limited, rounded proximal edges [2, 43]. A study by Ronger et al. found that cases of NAM were significantly associated with a brown coloration of the background, and the presence of irregular longitudinal lines (per color, spacing, thickness, and parallelism) (Figure 9) [3, 43]. The study also observed the micro-Hutchinson's sign (a Hutchinson's sign that is too small to be seen with the human eye) only in cases of melanoma [3, 43]. However, the micro-Hutchinson's sign rarely occurs, and the study was unable to statistically evaluate for specificity [3, 43]. Nail apparatus nevi were significantly associated with a brown coloration of the background and the presence of regular lines, while nail apparatus lentigines, ethnic-type pigmentation, and drug-induced pigmentation were significantly associated with homogeneous longitudinal thin gray lines and gray coloration of the background [3, 43].

Additionally, dermoscopy of the free edge of the nail plate may facilitate preoperative mapping by helping to identify the origin of the pigmented band in the matrix [3, 45]. A pigmented band in the ventral nail plate originates from the distal matrix, whereas a band in the dorsal nail plate originates in the proximal matrix [3, 45]. If despite dermoscopic evaluation, doubt remains as to the location of the pigmented band in the matrix, a distal nail clipping stained with Fontana Masson will demonstrate the matrix band origin [3].

**ABC Rule for Clinical Detection of NAM**

An ABCDEF mnemonic was created to help both clinicians recall certain key clinical features that should raise suspicion for the possibility of subungual melanoma [46]. “**A**” stands for the *age* of the patient at presentation [46]. Subungual melanoma occurs most commonly within the 5th–7th decades, although it has been reported to occur in patients as young as 1 year old and as old as 90 years [46]. “***B***” represents the most common clinical presentation of a subungual melanoma with a pigmented band composed of variegated shades of *brown-to-black* with a *breadth* of over 3 mm and irregular or *blurred borders [46]*. “***C***” is for *change*—a sudden, recent, or rapid increase in the size of the pigmented band is considered comparable to the radial growth phase [46]. A change in nail plate morphology is also concerning for melanoma [46]. “***D***” signifies single *digit* involvement with the thumb, then great toe or index finger being most commonly involved [46]. “***E***” denotes the *extension* of pigment into the perionychium in association with melanoma, or Hutchinson's sign [46]. “***F***” stands for *family* or personal history of melanoma and/or dysplastic nevus syndrome [46].

## 4. Biopsy of Melanonychia

As the clinical diagnosis of melanonychia is frequently difficult, a biopsy is oftentimes necessary to rule out melanoma [3]. In fact, a study by Di Chiacchio et al. found that the overall accuracy of dermatologists in the preoperative diagnosis of NAM *in situ* was low ranging from 46–55% [47]. Interestingly, the study also found that a dermatologist's level of expertise in nail disease did not statistically influence the correct diagnosis [47]. 

While no formally adopted algorithm with guidelines for when to perform a diagnostic biopsy exists, there are several suggested clinical practices that help guide clinical decision making [4]. The threshold for biopsy should be low in a white patient with unexplained melanonychia of a single digit [2, 46]. For cases of unexplained melanonychia of a single digit in a nonwhite patient, or of multiple digits in a patient with any phototype, melanonychia should be closely monitored and biopsied if any suspicious features arise [2, 46]. Because unexplained melanonychia in children is rarely due to an underlying melanoma, their management is more conservative [2, 47].

When performing a biopsy, the origin of the pigment, which most often lies in the nail matrix, should be sampled in its entirety. The nail matrix biopsy, while a relatively safe and simple procedure when basic principles are followed, is associated with the greatest risk in terms of scarring when compared to other biopsy locations within the nail unit [48]. With regards to the anatomy and physiology of the nail unit, the distal matrix forms the ventral nail plate, and the proximal matrix forms the dorsal nail plate [4, 48–51]. Thus a biopsy of the distal matrix is almost always preferred over a proximal matrix biopsy as any resultant scar, which clinically manifests as a thinned ridge, would lie on the undersurface of the nail plate [4, 48–51]. Thinned ridges on the dorsal surface of the nail plate are easily traumatized as they catch on items such as clothing and are much more troublesome for the patient [4].

In order to minimize any potential scarring, nail matrix excisional biopsies can be oriented transversely [3, 4, 48]. Moreover, full thickness nail matrix biopsies larger than 3 mm can be sutured in order to achieve an optimal cosmetic result [1, 52].

The punch biopsy is generally reserved for longitudinal melanonychia less than 2.5–3 mm in width originating in the distal matrix [1, 3]. A punch biopsy should be taken at the origin of the pigmented band and should extend to depth of the periosteum [3, 48]. Punch biopsies are generally not recommended for evaluating pigmented lesions 3 mm or more in width (even if multiple punch biopsies are taken) because peripheral pigmentation may not be adequately sampled to rule out malignancy. Additionally, taking serial punch biopsies is associated with an increased risk of permanent nail dystrophy [3].

The lateral longitudinal excision is best suited for biopsying suspicious lesions located in the lateral one-third of the nail as this technique samples all components of the nail unit including the nail matrix, nail bed, nail fold, and hyponychium [1, 3]. The tangential (shave) excision is ideal for sampling a longitudinal pigmented band with a lower preoperative suspicion of melanoma that is greater than 3 mm in width in the midnail plate or of any width originating in the proximal matrix [1, 3, 53]. The tangential technique, first described by Eckart Haneke in 1999, is less invasive than a transversely-oriented matrix excision, and it is associated with minimal long-term dystrophy despite the increased width commonly associated with its use [1, 3, 53]. However, for any case with a high preoperative likelihood of invasive melanoma, a full-thickness nail matrix biopsy is necessary for prognosis determination as a tangential biopsy may not provide an accurate Breslow depth [3].

## Figures and Tables

**Table 1:** Classification of conditions associated with longitudinal melanonychia. Table adapted from J. Andre, N. Lateur. Pigmented nail disorders [2].

Melanonychia
Melanocytic activation

Physiologic causes
Racial melanonychia
Pregnancy

Local and regional causes
Repeated local trauma from poor footwear or overriding toes
Onychotillomania
Nail biting
Occupational trauma
Carpal tunnel syndrome

Dermatologic causes
Onychomycosis
Chronic paronychia
Psoriasis
Lichen planus
Amyloidosis
Chronic radiation dermatitis
Systemic lupus erythematosus
Localized scleroderma
Onychomatricoma
Bowen's disease
Myxoid pseudocyst
Basal cell carcinoma
Subungual fibrous histiocytoma
Verruca vulgaris
Subungual linear keratosis

Systemic causes
Endocrine disorders (Addison's disease, Cushing's syndrome,
Nelson's syndrome, hyperthyroidism, and acromegaly)
Alcaptonuria
Nutritional disorders
Hemosiderosis
Hyperbilirubinemia
Porphyria
Graft versus host disease (lichen planus-type changes
accompanied by longitudinal melanonychia)
AIDS

Iatrogenic causes
Phototherapy
X-ray exposure
Electron beam therapy
Drug intake^∗^—please see Table 2

Syndromes
Laugier-Hunziker syndrome
Peutz-Jegher syndrome
Touraine syndrome

Melanocytic hyperplasia

Lentigo

Nevus
Congenital nevi
Acquired nevi

Nail apparatus *Insitu* and invasive melanoma

**Table 2:** Drugs associated with melanocytic activation and subsequent longitudinal melanonychia. Table adapted from J. Andre, N. Lateur. Pigmented nail disorders [2].

Drug-induced melanonychia	
Chemotherapeuticals	Others

Bleomycin sulfate	ACTH
Busulfan	Amodiaquine
Cyclophosphamide	Amorolfine
Dacarbazine	Arsenic
Daunorubicin hydrochloride	Chloroquine
Doxorubicin	Clofazimine
Etoposide	Clomipramine
5-fluorouracile	Cyclones
Hydroxyurea	Fluconazole
Imatinib	Fluorides
Melphalan hydrochloride	Gold salts
Methotrexate	Ibuprofen
Nitrogen mustard	Ketoconazole
Nitrosourea	Lamivudine
Tegafur	Mepacrine
	Mercury
	MSH
	Minocycline
	PCB
	Phenytoin
	Phenothiazine
	Psoralen
	Roxithromycin
	Steroids
	Sulfonamide
	Thallium
	Timolol
	Zidovudine
